# Clinial Features, Individualized Treatment and Long-Term Surgical Outcomes of Skull Base Meningiomas With Extracranial Extensions

**DOI:** 10.3389/fonc.2020.01054

**Published:** 2020-06-30

**Authors:** Houjie Liu, Haipeng Qian, Xueji Li, Fuxing Zuo, Xiaoli Meng, Shaoyan Liu, Jinghai Wan

**Affiliations:** ^1^Department of Neurosurgery, National Cancer Center/National Clinical Research Center for Cancer/Cancer Hospital, Chinese Academy of Medical Science and Peking Union Medical College, Beijing, China; ^2^Department of Head and Neck Surgery, National Cancer Center/National Clinical Research Center for Cancer/Cancer Hospital, Chinese Academy of Medical Science and Peking Union Medical College, Beijing, China

**Keywords:** clinical features, craniofacial, Ki-67, prognostic factors, progression-free survival, radiotherapy, skull base meningioma

## Abstract

**Object:** Skull base meningiomas with extracranial extensions are rarely described. This study describes the clinical features, surgical management and clinical outcomes of these rare tumors and investigates risk factors associated with progression-free survival (PFS).

**Methods:** The clinical data of 34 consecutive patients who underwent surgery for skull base meningiomas with extracranial extensions from 2007 to 2018 were retrospectively collected and analyzed.

**Results:** The mean patient age was 47.9 ± 13.9 years; 50.0% were male. The most common symptoms on admission were ophthalmic. All patients underwent a multidisciplinary consultation before surgery, and received individualized surgical management. The gross total resection (GTR) rate was 55.9% (19/34). Twelve patients received post-operative adjuvant radiotherapy (RT). Twelve patients experienced tumor recurrence during the follow-up period. The median PFS duration was 54 months. The mean overall survival (OS) duration was 111 months. By univariate analysis, a higher histological grade (WHO grade II and III), Ki-67 LI ≥ 5 and the extent of resection (EOR) were significantly associated with tumor recurrence. Multivariate analysis revealed Ki-67 LI ≥ 5, the EOR and adjuvant RT as prognostic factor of PFS.

**Conclusions:** These relatively rare meningiomas are difficult to resect and have a poor prognosis; they are more common in males and have a higher histological grade than intracranial meningiomas. Multidisciplinary collaboration and individualized surgical strategies are crucial for surgically managing these complex tumors. Total removal of the tumor remains challenging. Subtotal resection (STR) or partial resection (PR) followed by RT is a reasonable strategy when radical resection is infeasible. Adjuvant RT should be recommended especially for tumors with histopathological risk factors (Ki-67 LI ≥ 5 or high histological grade).

## Introduction

Intracranial meningiomas are extracerebral, slow-growing, well-defined tumors that account for 13–26% of all primary intracranial neoplasms, and ~25% of meningiomas arise in the cranial base (1). World Health Organization (WHO) grade II and III meningioma, ~10–20% of all intracranial meningioma, exhibit a more aggressive biological behavior and a greater probability for recurrence than WHO grade I (WHO-I) meningioma (2).

Skull base meningiomas with extracranial extensions are a relatively rare clinical entity which extend into craniofacial structures (3). These complex tumors involve both intracranial and extracranial structures, such as the anterior or middle cranial fossa and the infratemporal fossa, nasal cavity, paranasal sinuses, orbits or neck, etc., so multidisciplinary cooperation and individualized surgical strategies are particularly required (4). A higher proportion of recurrent and non-benign tumor have been found in patients with such a special skull base meningioma (5), and RT is often needed after surgery. So, it is difficult to surgically manage this special entity, especially for a single disciplinary team. To the best of our knowledge, only a few reports have discussed this rare subtype of meningioma with consecutive patient series (3–16). Furthermore, most of them focused on the evaluation of sphenoorbital meningiomas or the outcome of a specific surgical approach. Individualized surgical approaches and the importance of multidisciplinary cooperation has not been highlighted. The clinical features and prognosis of these patients with skull base meningiomas with extracranial extensions, as well as comprehensive treatment strategies, have not yet been systematically well-documented.

Multidisciplinary cooperative treatment strategies for intra- and extra-cranial communicating tumors of the skull base have been explored at the Cancer Hospital, Chinese Academy of Medical Science and Peking Union Medical College, and very good results have been achieved for dumbbell shaped jugular foramen schwannomas with neck extension surgically treated since 2005 (17, 18). In this study, we retrospectively analyzed the cases with pathologically confirmed skull base meningiomas with extracranial extensions that received multidisciplinary treatment at our center from 2007 to 2018, with focus on clinical features, individualized management paradigm and prognosis analysis.

## Materials and Methods

### Patient Population

From 2007 to 2018, 271 patients with skull base meningiomas were surgically treated in the Department of Neurosurgery, Cancer Hospital, Chinese Academy of Medical Science and Peking Union Medical College. Of these, 34 patients with skull base meningiomas with extracranial extensions were included. Twenty-nine patients underwent one operation, four patients underwent twice operations, and one underwent four operations for their recurrent tumors, and a total of 41 operative procedures had been performed in the series. The diagnosis of meningioma was confirmed by the neuropathologist according to either the 2007 or 2016 WHO grading criteria in all cases. The study was approved by the Cancer Hospital, Chinese Academy of Medical Science and Peking Union Medical College Research Ethics Committee. Written consent from patients that are identifiable from the images have been obtained.

### Clinical and Radiological Data

Clinical and radiological data were collected and analyzed. Clinical data included patient's age on admission, sex, and clinical manifestations (e.g., visual impairment, proptosis, headache, and neurological deficits). All patients underwent computed tomography (CT) and magnetic resonance imaging (MRI) scans during the diagnostic workup. High-resolution CT imaging with bone-window algorithm provided the best images of hyperostosis or erosion of the bone structure of the skull base. Gadolinium (Gd)-enhanced MRI sequence was used to confirm the intracranial and extracranial tumor portion and to evaluate the degree of dura involvement. Gd-enhanced MRIs were classified into two groups, including heterogeneous and homogeneous enhancement.

### Pathological Examination

After operation, the dural attachment and areas of involved bone, nerve, skeletal muscle and mucosa were sent for pathological examination. Fresh paraffin-embedded tumor tissue was stained with hematoxylin and eosin (H&E) and immunohistochemistry for diagnosis and differential diagnosis. The ki-67 label index (Ki-67 LI) is the percentage of cells reactive to Ki-67, and the cutoff values for the Ki-67 LI were defined as 5% based on the results of our data and previous reports (2, 19).

### Operative Procedures

Operative notes described the details of the surgical approach and the EOR. The surgical approach was selected depending upon the location of the tumor and the dimensions of its extracranial and intracranial components. Anterior skull base meningiomas with nasal extension were removed via a Derome approach. Sphenoid wing meningiomas with orbital extension were resected via a frontotemporal approach. Middle skull base meningiomas with infratemporal or pterygopalatine fossa extension were resected by a middle cranial fossa approach (the major part of tumor was intracranial) or a maxillary swing approach (the major part of tumor was extracranial). Midline skull base meningiomas with nasal extension were resected by a purely endoscopic endonasal approach. Dumbbell shaped jugular foramen meningiomas with neck extension were resected via a combined craniocervical approach. Cranial base meningiomas with extensive intra- and extra-cranial involvement were removed by a combined craniofacial approach or the undefined approach. The tumor resection was carried out according to the principle of microneurosurgery, that is, to devascularize the tumor first, to debulk the tumor, and then to remove the capsule and involved dura and bone. En bloc removal of the tumor is appreciated if possible. Skull base defects following by tumor resection were all reconstructed. During the operation, the neurosurgeons were mainly responsible for the craniotomy, intracranial tumor resection, skull base repair, and skull closure. The head and neck surgeons were mainly responsible for ligation of the external carotid artery or its branches, transfacial approach, extracranial tumor resection and free or pedicled myocutaneous flap transplantation. Based on a review of the surgery records, the EOR was subdivided into GTR, STR and PR. In general, GTR could be categorized as Simpson grade I or II, STR could be categorized as Simpson grade III or IV, and PR could be categorized as Simpson grade IV with significant residual tumor or V (20).

### Follow-Up

Follow-up notes were collected and analyzed. Disease progression, defined as tumor recurrence after GTR or residual tumor enlargement after STR or PR, was evaluated using enhanced MRI scans. Complications, post-operative adjuvant RT, OS, and PFS were also recorded.

### Statistical Analysis

Continuous variables were expressed as mean ± standard deviation with a range. Categorical variables were described using frequencies and percentages. OS was determined from the date of surgery to death or the last follow-up. PFS was determined from the date of surgery to the date of documented progression. To assess predictors of PFS, we examined the following items: age, sex, lesion recurrence, enhancement on Gd-enhanced MRI, histological grade, Ki-67 LI, EOR, and adjuvant RT. The rates of PFS were estimated by Kaplan-Meier analysis (log-rank test). The Cox proportional hazards model and a stepwise regression analysis were used to assess the relevance between factors and recurrence. A *P* < 0.05 was considered significant. Statistical analyses were performed using SPSS Statistics software (version 21.0; IBM Corp). It is noteworthy that one patient who died during the perioperative period was not included in the Kaplan-Meier analysis because he did not meet the purpose of the study and his death cannot reflect the natural course of this tumor.

## Results

### Epidemiological and Clinical Data

Thirty-four cases of pathologically confirmed skull base meningioma with extracranial extensions were identified among 271 cases of surgically treated skull base meningiomas in our center in the study period. Thus, the ratio of this subtype of skull base meningiomas was 12.55%. The clinical characteristics are summarized in Table 1. The average patient age was 47.9 ± 13.9 years (range, 14–72 years). Seventeen patients were female, accounting for 50.0% of all patients (male: female ratio, 1:1). The most common presenting symptoms were ophthalmic symptoms, such as visual impairments, proptosis or retro-orbital pain, occurring in 21 patients (61.8%). Other tumor manifestations on admission were cranial nerve disorders in 9 patients (26.5%), a mass on the face or neck in 8 (23.5%), nasal obstruction or discharge in 4 (11.8%), and headache in 4 (11.8%). The mean duration of symptoms before surgery was 19.2 months (range, 7 days-10 years). Fourteen patients were initially treated in our center, and the other 20 patients with recurrent tumor had been surgically treated elsewhere before admission to our center.

**Table 1:** Demographic, clinical, and radiological characteristics of 34 patients with skull base meningiomas with extracranial extensions.

**Characteristics**	**Value (%)**	**Characteristics**	**Value (%)**
Age at diagnosis (years)		Enhancement	
Mean	47.9 ± 13.9	Homogeneous	26(76.5)
Range	14–72	Heterogeneous	8(23.5)
Sex		Bone structure change on CT	22(64.7)
Male	17 (50.0)	Pathology	
Female	17 (50.0)	WHO grade I	20(58.8)
Duration from onset to admission		WHO grade II	12(35.3)
Mean	19.2	WHO grade III	2(5.9)
Range	7 days-10 years	Ki-67 LI	
Presenting symptoms		≥5%	17(50.0)
Headache	4 (11.8)	<5%	17(50.0)
Ophthalmic symptoms	21 (61.8)	EOR	
Mass on face or neck	8 (23.5)	GTR	19(55.9)
Nasal obstruction or discharge	4 (11.8)	STR	9(26.5)
Cranial nerve disorders	9 (26.5)	PR	6(17.6)
Initial or recurrent lesion		Adjuvant RT	
Initial	14 (41.2)	Yes	12(35.3)
Recurrent	20 (58.8)	No	21(61.8)
Extracranial extensions		NK	1(2.9)
Orbit	30 (88.2)	Tumor recurrence	12(35.3)
Nasal cavity or paranasal sinus	20 (58.8)	Postoperative death	5(14.7)
Infratemporal or pterygopalatine fossa	8 (23.5)	Died in perioperative period	1(2.9)
Neck or parapharyngeal	3 (8.8)	Died in follow-up period	4(11.8)
space		Median follow-up duration (months)	31

*LI, label index; NK, not known; RT, radiotherapy*.

### Radiological Features

**Figure 1:**
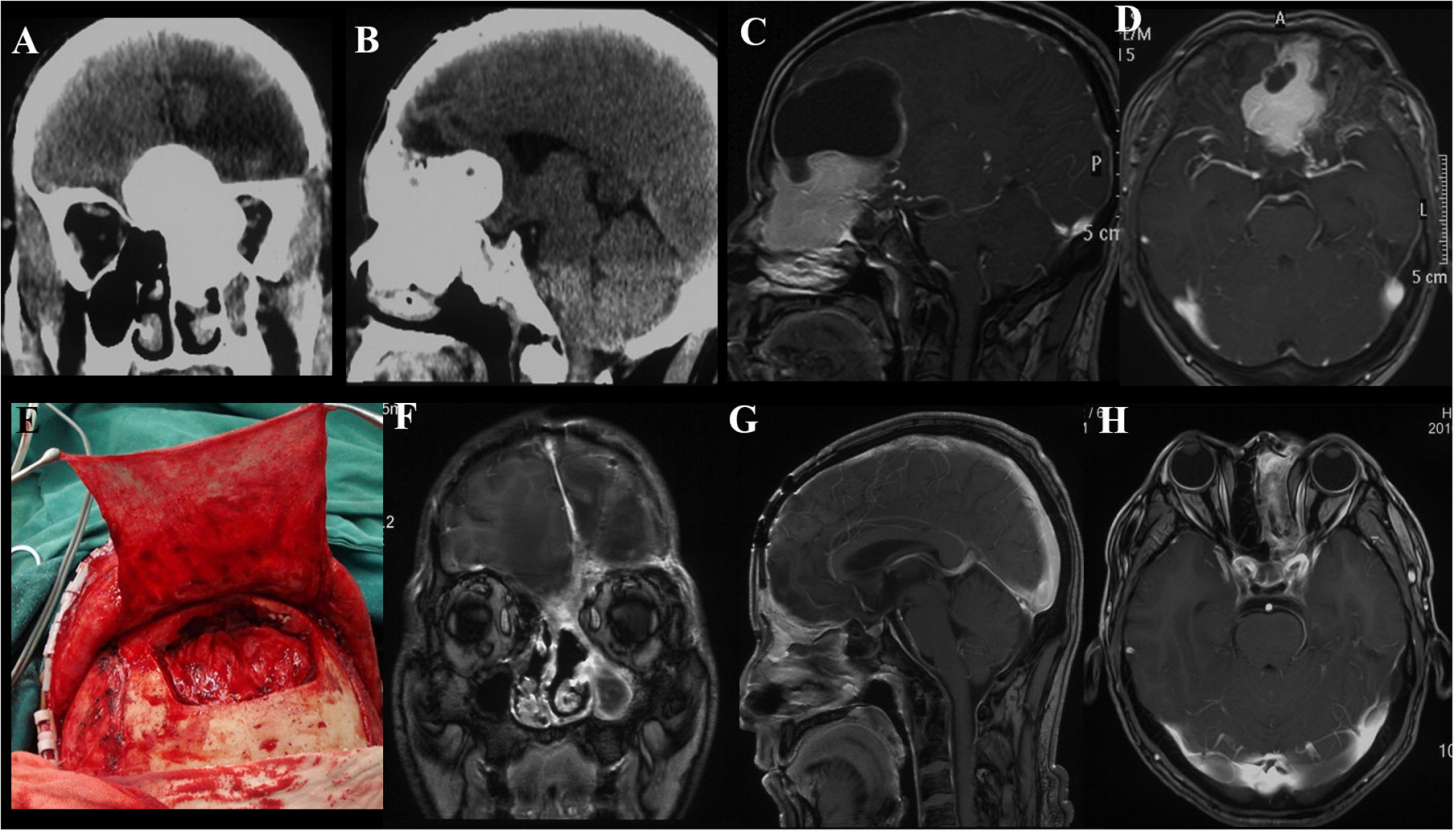
Case illustrating the Derome approach. **(A,B)** CT scans show an anterior skull base tumor with nasal extension with significant calcifications. **(C,D)** T1-weighted sagittal and axial contrast-enhanced MRIs show the same tumor as A and B, the irregularly shaped lesion with heterogeneous enhancement and cystic changes. **(E)** A periosteal flap was prepared for repairing the skull base defect. **(F–H)** Postoperative contrast-enhanced MRIs indicated GTR of the tumor.

**Figure 2:**
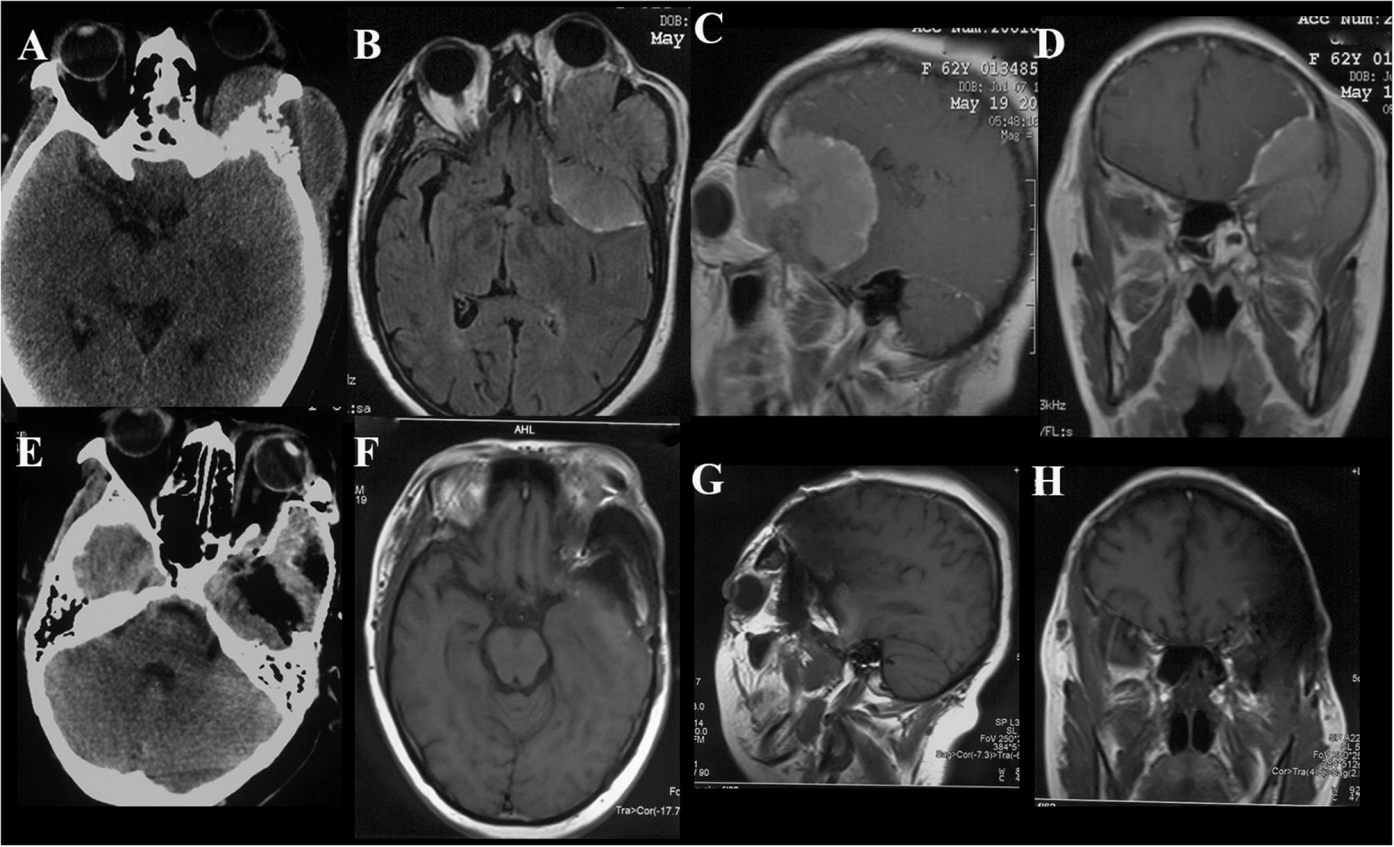
Case illustrating the frontotemporal approach. **(A)** CT scan show bone hyperostosis and destructive absorption of the left sphenoidal wing and lateral wall. **(B–D)** T1-weighted axial, sagittal and coronal contrast-enhanced MRIs demonstrate the same tumor as A, the anterior and lateral cranial base meningioma with orbital extension with homogeneous enhancement. **(E–H)** Postoperative CT and MRI indicate satisfactory tumor resection.

**Figure 3:**
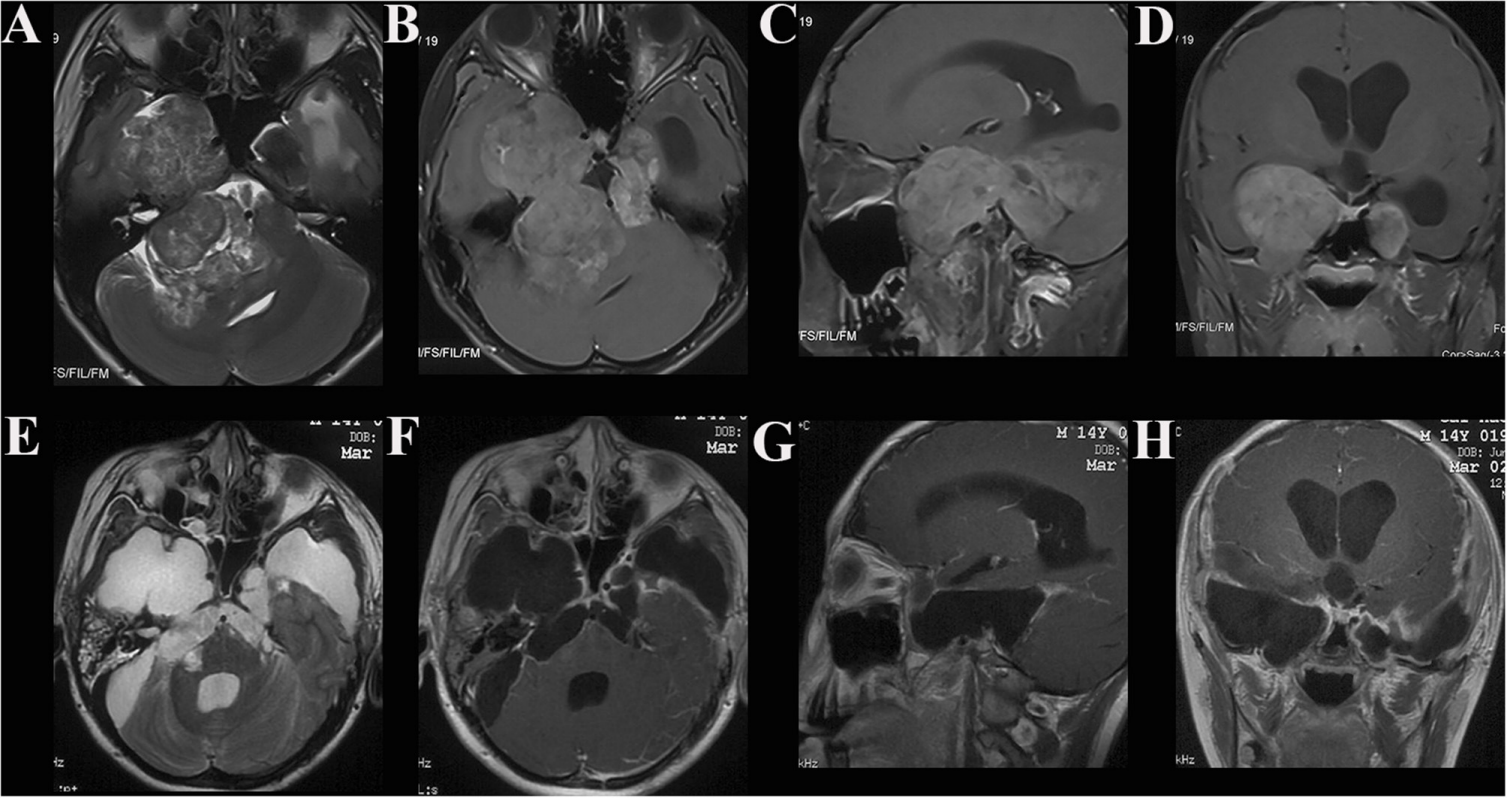
Case illustrating the middle cranial fossa approach. **(A)** T2-weighted axial MRI show dumbbell shaped tumors in the bilateral middle and posterior cranial base with hyperintense and isointense. **(B–D)** T1-weighted axial, sagittal, and coronal contrast-enhanced MRIs demonstrate both tumors with heterogeneous enhancement, the dumbell shaped tumor on the right extended into infratemporal fossae **(B)**. **(E–H)** Postoperative contrast-enhanced MRI indicated satisfactory tumor resection.

MRI or CT scans were used for the imaging diagnosis of meningioma and the evaluation of tumor resection. In this study, tumors often invaded different extracranial structures at the same time. Skull base meningiomas extended into orbital regions in 30 patients (88.2%), extended into the nasal cavity and/or paranasal sinuses in 20 (58.8%), invaded the infratemporal fossa or pterygopalatine fossa in 8 (23.5%), and involved the neck or parapharyngeal space in 3 (8.8%). Isointense or slightly hypointense on T1-weighted MRI and hyperintense or isointense on T2-weighted MRI were presented in most tumors. Gd-enhanced MRIs demonstrated significant enhancement in all patients, including heterogeneous enhancement in 10 cases (29.9%) and homogenous enhancement in 24 cases (70.1%). Tumor calcification was found on CT scans in 3 patients (8.8%) (Figures 1A,B). Cystic degeneration was revealed on MRI in 3 patients (8.8%) (Figure 1C). Distinct dural tail sign was found on enhanced MRI in 10 patients (29.4%) (Figure 2), whereas was not found in the other 24 (Figure 3). Changes in the bone structure of the skull base were found on CT scans with the bone-window algorithm in 22 patients (64.7%), including hyperostosis (63.6%) (Figure 2A) and destructive absorption (36.4%) (**Figure 5D**). In most patients, the bone hyperostosis areas were resected, as verified by post-operative CT. Fluid-attenuated inversion recovery imaging confirmed the existence of edema surrounding the tumor. Four patients in this series underwent preoperative angiography and embolization due to abundant tumor blood supply.

### Surgical Records and Complications

**Figure 4:**
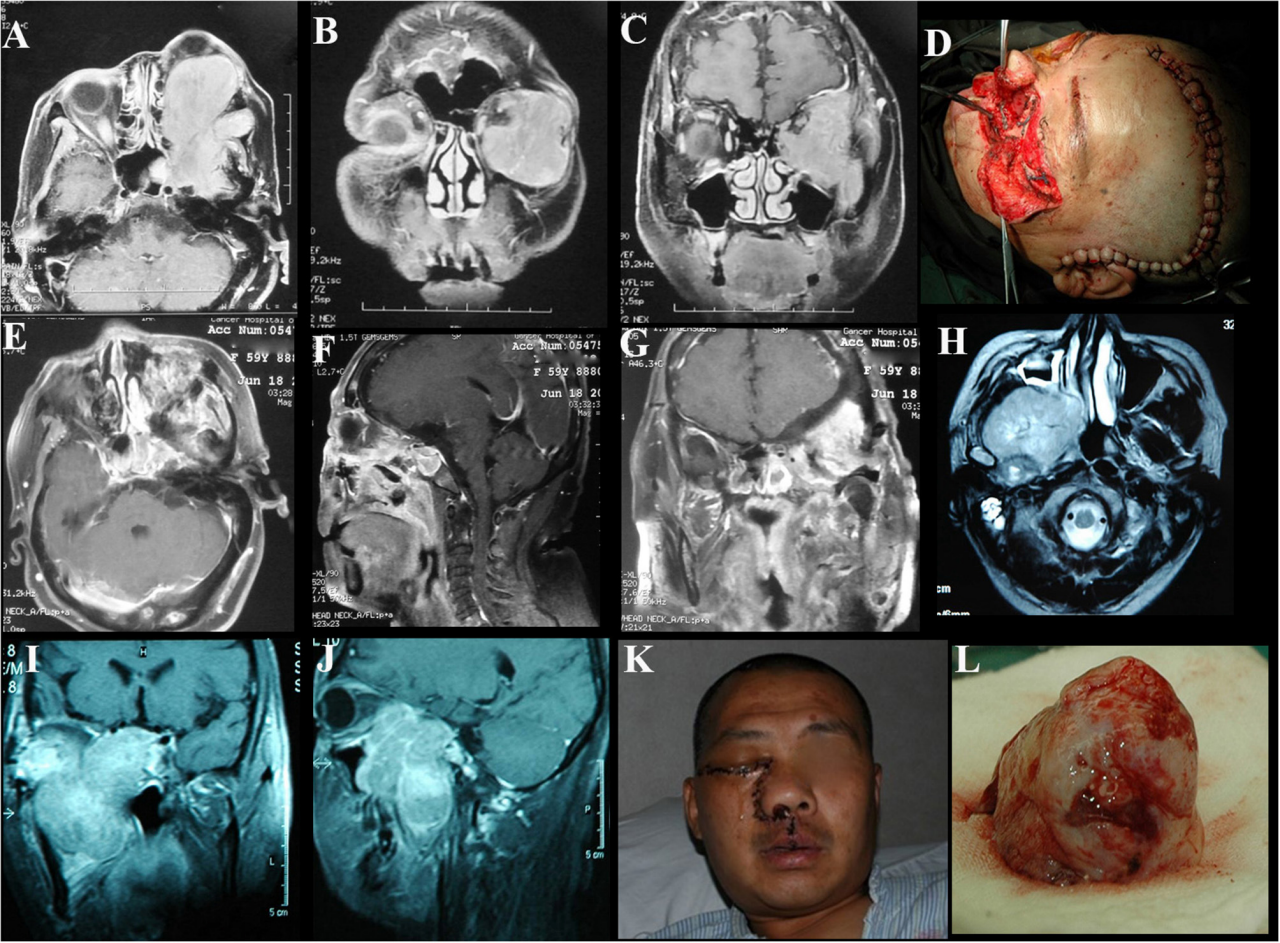
Case illustrating the craniofacial and maxillary swing approaches. **(A–C)** T1-weighted axial and coronal contrast-enhanced MRIs demonstrate a tumor with homogeneous enhancement located in the anterior and lateral cranial base with extension to the orbits and infratemporal fossa. **(D)** The incision of the craniofacial approach. **(E–G)** Contrast-enhanced MRIs 1 week after the surgery indicate satisfactory tumor resection. **(H–J)** T1-weighted axial, coronal, and sagittal contrast-enhanced MRIs demonstrate a middle skull base meningioma extended into the right infratemporal and pterygopalatine fossae with significant enhancement. **(K)** The incision of the maxillary swing approach. **(L)** Photograph of the tumor sample. The maxillary swing approach can provide wide exposure and allow en bloc tumor resection.

**Figure 5:**
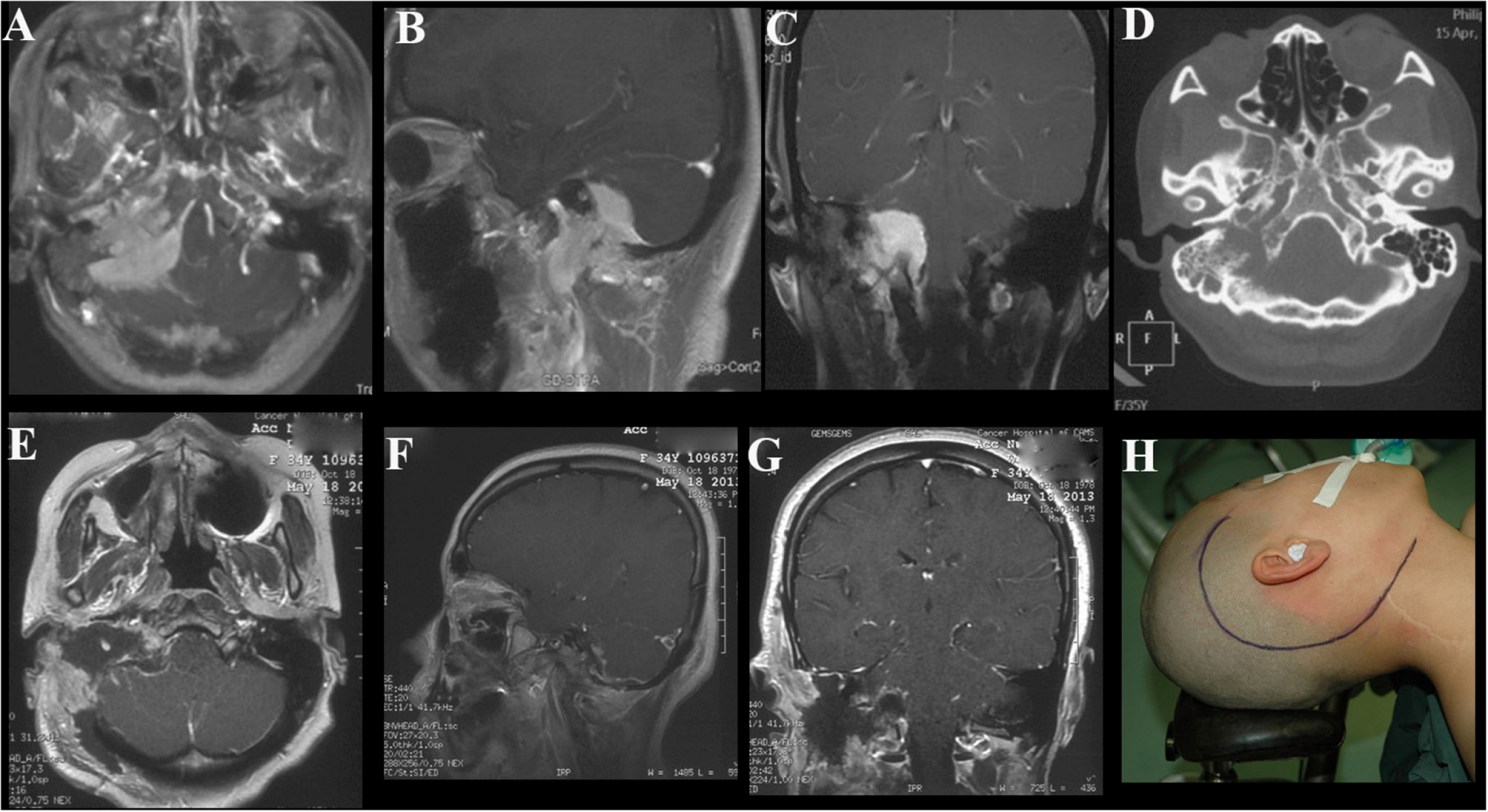
Case illustrating the combined craniocervical approach. **(A–C)** T1-weighted contrast-enhanced MRIs demonstrate a tumor with homogeneous enhancement located in the right jugular foramen with both posterior cranial fossa and neck extensions, dural tail signs are found in the posterior fossa. **(D)** CT scan with the bone-window algorithm shows the enlarged jugular foramen with bone destructive change. **(E–G)** Postoperative contrast-enhanced MRIs indicate satisfactory tumor resection. **(H)** The incision of the combined craniocervical approach.

**Figure 6:**
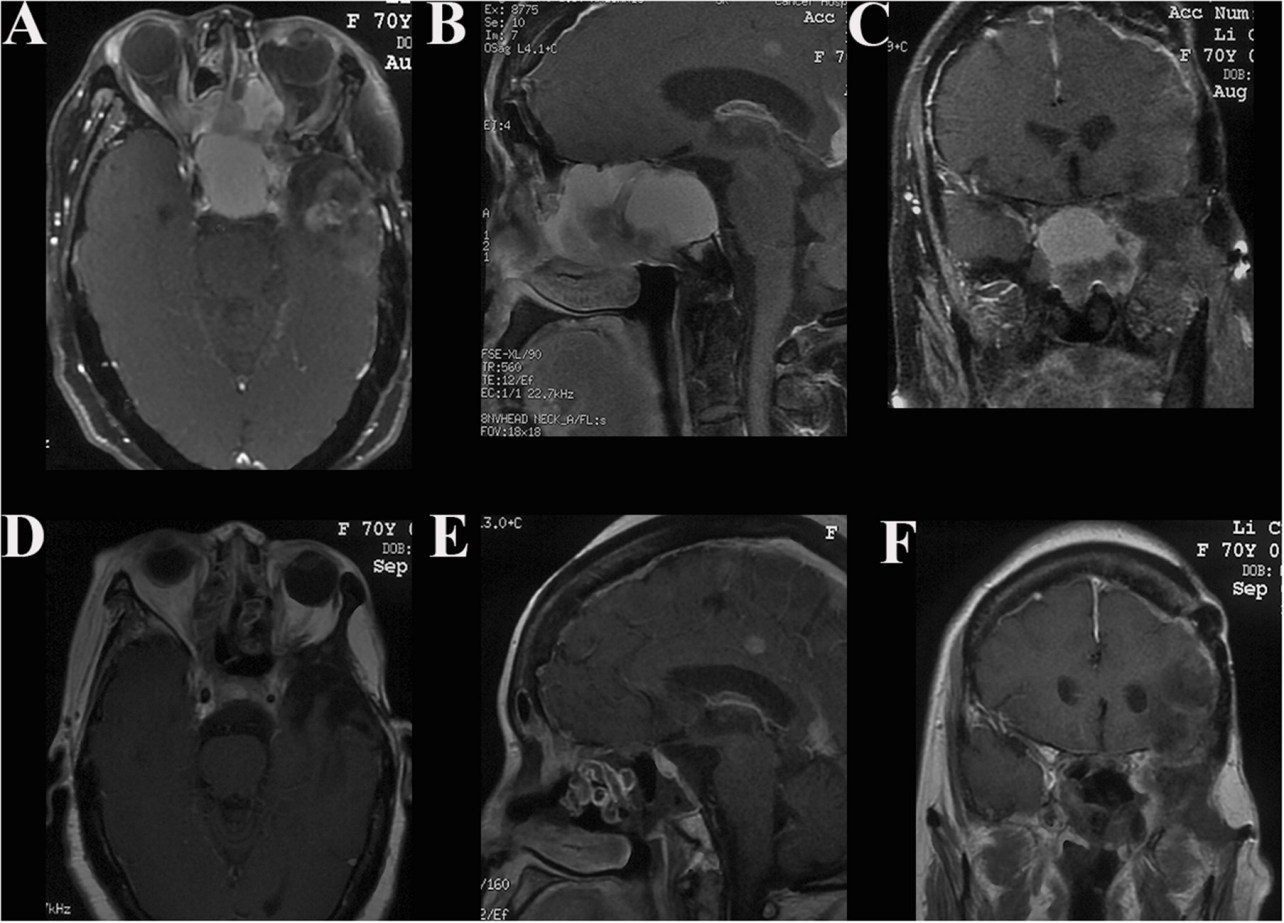
Case illustrating the endoscopic endonasal approach. **(A–C)** T1-weighted axial, sagittal, and coronal contrast-enhanced MRIs demonstrate an anterior skull base tumor with extension to the sphenoid sinus, ethmoidal sinus, and nasal cavity with moderate enhancement. **(D–F)** Postoperative contrast-enhanced MRIs indicate satisfactory tumor resection.

**Figure 7:**
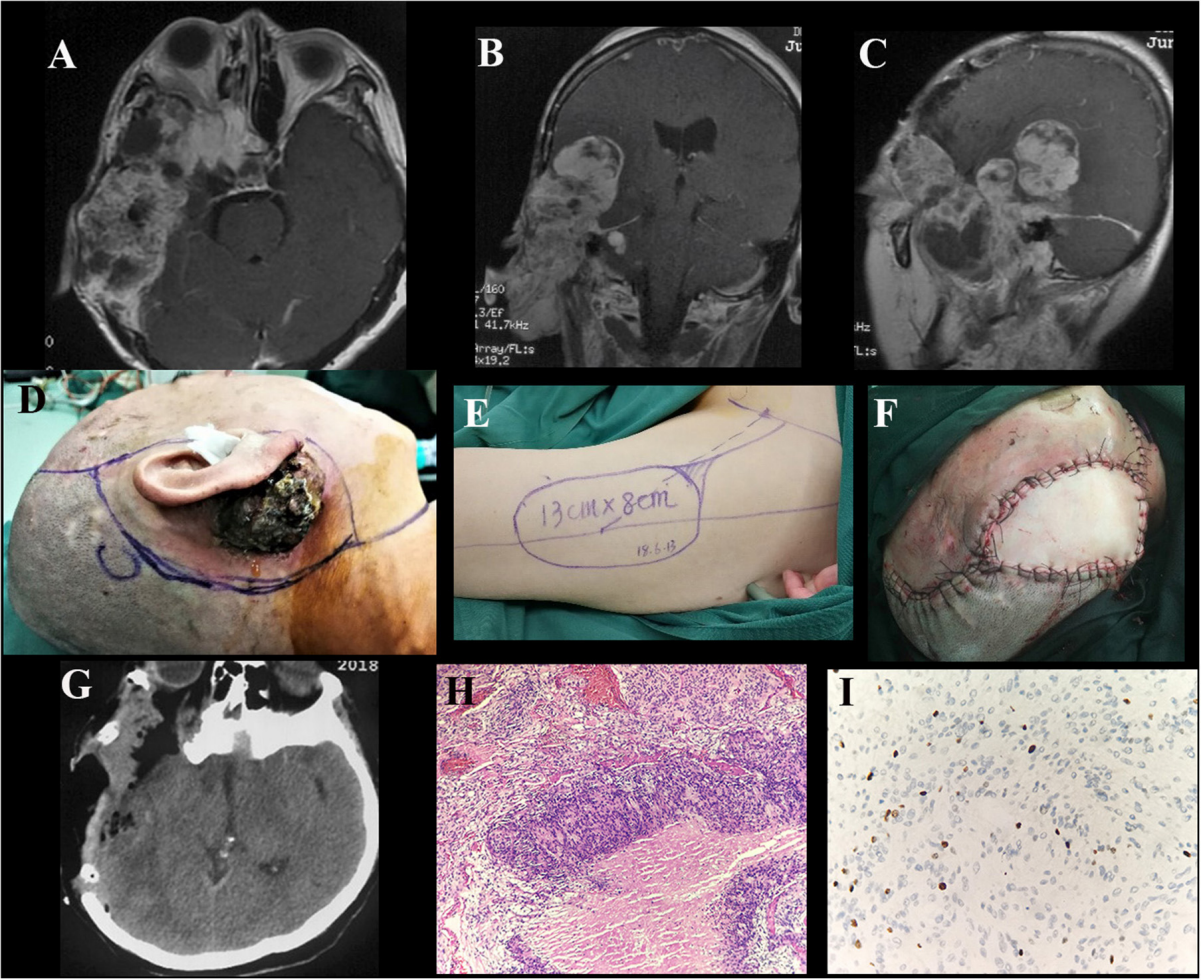
Case illustrating an undefined approach. **(A–C)** T1-weighted axial and coronal contrast-enhanced MRIs demonstrate an irregularly shaped tumor originating in the right middle skull base with both intra- and extra-cranial extensions, invading the right ear with skin ulceration. The tumor enhanced heterogeneously after injection of contrast agent. **(D–F)** The incision surrounding the lesion and the free flap from the anterolateral thigh harvested to repair the defect. **(G)** Postoperative CT scan shows tumor and bone involvement resection. **(H)** H&E × 100 indicate atypical meningioma (WHO grade II). **(I)** Immunohistochemical staining shows Ki-67 ≥ 5.

All operations were performed by neurosurgeons, with the assistance of head and neck surgeons, if necessary. In all cases, therapies were tailored to individual patient after a multidisciplinary consultation. Eight patients underwent treatment by the Derome approach (Figure 1). In 1 of those 8 patients, a transnasal endoscopic approach was additionally required for the resection of tumors located in the ethmoid sinus and nasal cavity. Twelve patients were treated with the frontotemporal approach (Figure 2) and middle cranial fossa approach (Figure 3), three with the maxillary swing approach (Figures 4H–L), three with the combined craniocervical approach (Figure 5) and two with the purely endoscopic endonasal approach (Figure 6). A combined craniofacial approach (Figures 4A–G) was employed in 4 cases, and an undefined approach (Figures 7A–G) in 2 cases. GTR was achieved in 19 patients (55.9%), STR in 9 (26.5%), and PR in 6 (17.6%).

Surgical morbidities occurred in 10 patients, with 1 case of mortality; the patient died of brain stem dysfunction due to tumor invasion 15 days after the operation. Postoperative complications included new cranial nerve deficit in 3 cases (8.8%), cerebrospinal fluid (CSF) leakage requiring temporary lumbar CSF drainage in 2 (5.9%), subcutaneous hydrops in 2 (5.9%), intracranial infection in 2 (5.9%), post-operative cerebral infarction in 1 (2.9%) and skin flap necrosis requiring repeat free pedicle flap transplantation in 1 (2.9%). Except for hemiplegia after cerebral infarction and permanent neurological deficits in two patients, other complications gradually improved within a few months.

### Histological Data

All tumors were verified as meningiomas on pathological examination. Among them, 20 tumors (58.8%) were classified as WHO grade I, 12 (35.3%) were WHO grade II, and 2 (5.9%) were WHO grade III (Figure 7H). The Ki-67 LI in 17 cases was ≥5% and <5% in the other 17 cases (Figure 7I). Among the cases of ki-67≥5, there were 5 cases of WHO-I, 10 of WHO-II and 2 of WHO-III. The PFS decreased remarkably at a Ki-67 LI of 5%, demonstrating that the cutoff value of the Ki-67 LI was suitable for the analysis of this study, as previously reported (2, 19).

### Follow-Up and Adjuvant RT

During the median follow-up period of 31 months (range, 3–133 months), 12 patients (35.3%) developed tumor recurrence, which occurred after an average of 19 ± 16.7 months (range, 3–54 months). The median PFS duration was 54 months (Table 2). The 3-, 5-, and 10-year PFS rate was 0.63, 0.47, and 0.47, respectively. Four patients died of recurrence during the follow-up period, and the mean OS duration was 111 months. The 3-, 5-, and 10-year OS rate was 0.87, 0.80, and 0.80, respectively. Twelve of 34 patients (35.3%) received adjuvant RT after surgical resection. Of the 12 patients who developed tumor recurrence during the follow-up period, 1 was treated by RT, and 5 were treated by repeat surgery and RT. One patient developed metastatic lung disease and received chemotherapy during the follow-up period.

**Table 2:** PFS and OS of 33^*^ meningioma patients.

**PFS and OS**	**Value**
3-, 5-, 10-PFS	0.63, 0.47, 0.47
PFS, median (months)	54
3-, 5-, 10-OS	0.87, 0.80, 0.80
OS, mean (months)	111

* *One case of perioperative death was not included in the statistical analysis*.

### Variables Associated With Recurrence

**Figure 8:**
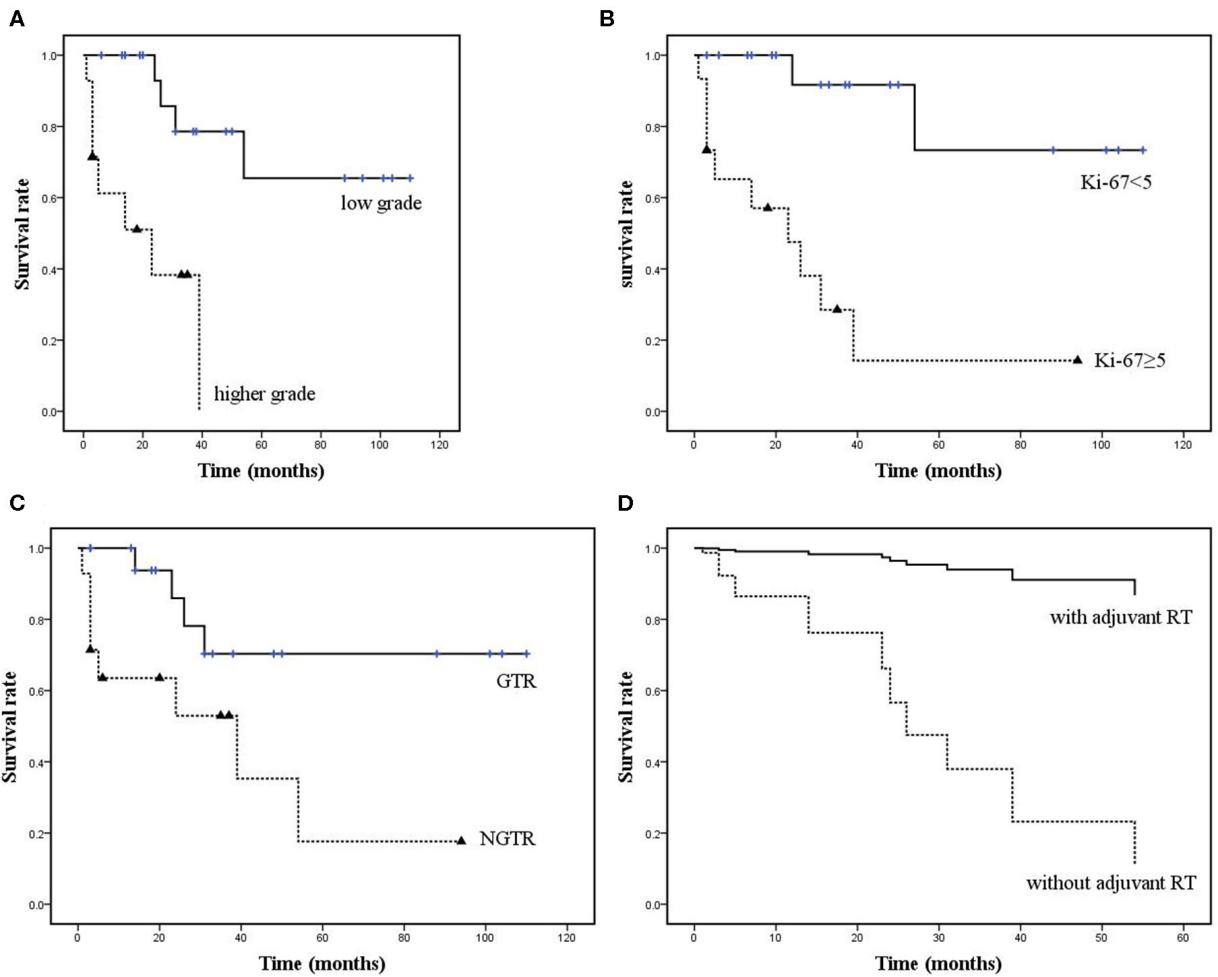
**(A–C)** Kaplan-Meier curves showing statistically significant differences in PFS based on histological grade, Ki-67 LI and EOR (*P* = 0.001, *P* < 0.001, and *P* = 0.024, respectively). **(D)** Adjuvant RT was also significantly associated with PFS on multivariate analysis (*P* = 0.024, HR: 15.632, 95% CI: 1.441–169.524).

**Figure 9:**
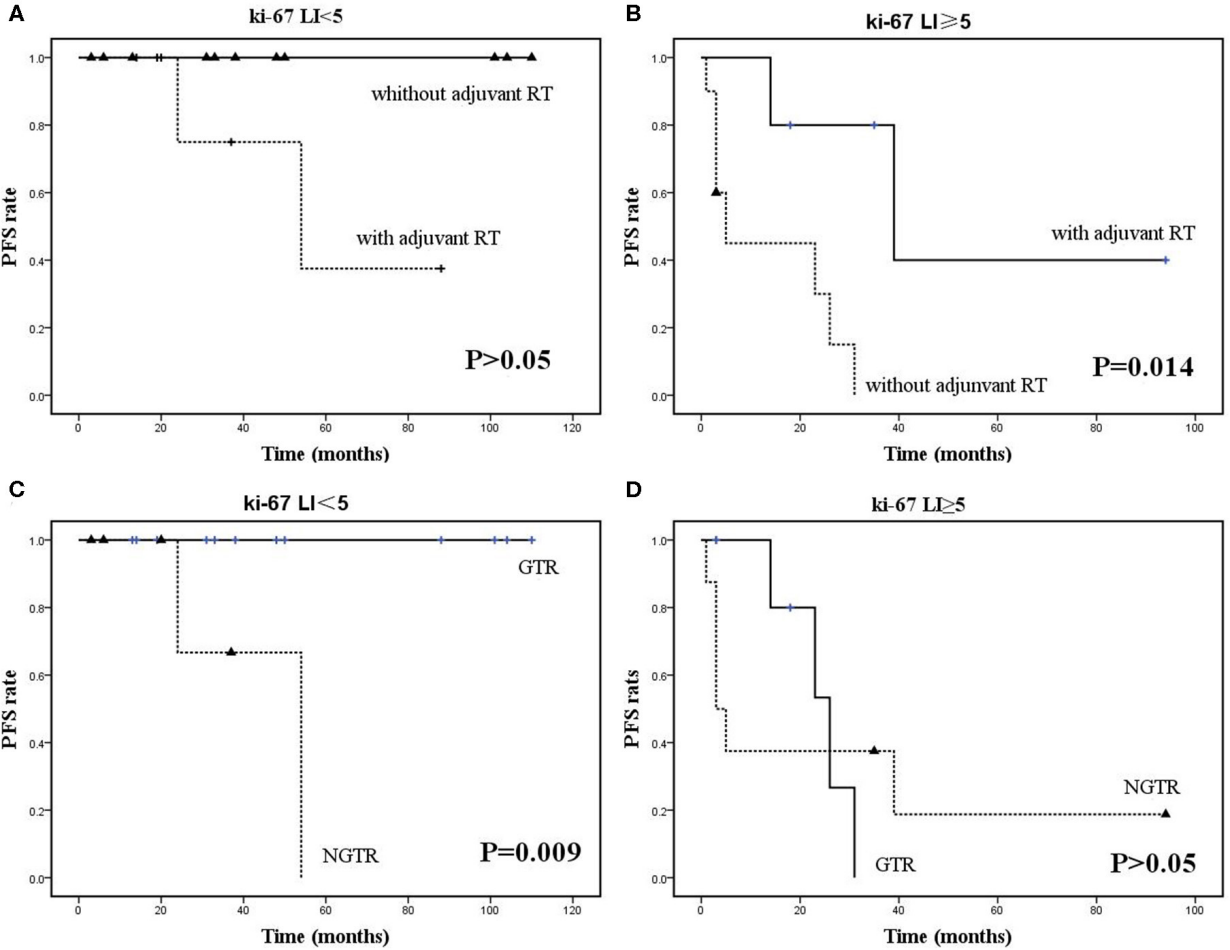
Kaplan-Meier survival curves. **(A,B)** Adjuvant RT was significantly associated with longer PFS in the Ki-67 LI ≥ 5 group but not in the Ki-67 LI <5 group. **(C,D)** GTR was significantly associated with longer PFS in the Ki-67 LI <5 group but not in the Ki-67 LI ≥ 5 group.

Age, sex, lesion recurrence, enhancement on Gd-enhanced MRI, histological grade, Ki-67 LI, EOR, and adjuvant RT were recorded and analyzed. Log-rank analysis and the Cox proportional hazards model were used to identify parameters significantly associated with shorter PFS. Univariate analysis revealed that a higher histological grade (Figure 8A), Ki-67 LI ≥ 5 (Figure 8B), and EOR (Figure 8C) were significantly associated with PFS, with *P* = 0.001, *P* < 0.001, and *P* = 0.024, respectively (Table 3). There was a trend toward increased recurrence in patients who were male (*P* = 0.097). Multivariate analysis confirmed Ki-67 LI≥5, EOR (not GTR; NGTR) and adjuvant RT (absent) (Figure 8D) as risk factors of shorter PFS. To identify whether adjuvant RT was necessary for NGTR, the PFS data were analyzed by dividing all patients into two groups: those with or without a high Ki-67 LI. In the Ki-67 LI ≥ 5 group, adjuvant RT was significantly associated with longer PFS, while it was not in the Ki-67 LI <5 group (Figures 9A,B). On the contrary, GTR was significantly associated with longer PFS in the Ki-67 group LI <5, while was not in the Ki-67 LI≥5 group (Figures 9C,D).

**Table 3:** Factors associated with PFS in 33 patients with skull base meningiomas with extracranial extensions.

**Variables**	**Univariate**	**Multivariate**	
	***P*-value**	***P*-value**	**HR (95% CI)**
Age (<50)	0.273	NA	
Sex (female)	0.097	0.159	0.375 (0.096–1.468)
Lesion recurrence	0.260	NA	
Heterogeneous enhancement	0.564	NA	
Higher histological grade	0.001^*^	0.37	2.314 (0.37–14.46)
Ki-67 LI ≥ 5	<0.001^*^	0.008^*^	9.774 (1.789–53.387)
EOR (NGTR)	0.024^*^	0.038^*^	10.937 (1.147–104.314)
Adjuvant RT (absent)	0.556	0.024^*^	15.632 (1.441–169.524)

* *P < 0.05; HR, hazard ratio; EOR, extent of resection; NGTR, not GTR; NA, not applicable*.

## Discussion

Research on meningiomas has never been popular. One reason could be the misunderstanding that these tumors are benign and curative. Neurosurgeons, especially skull base neurosurgeons, know this understanding to be wrong. Meningiomas have varying biological behaviors that range from completely benign to malignant. Moreover, even completely benign tumors occurring at the skull base are challenging to remove safely and can recur quickly (21).

### Clinical and Radiological Characteristics

The mean age of the cohort was 47.9 years, slightly lower than 48.3–64 years reported in the literature (5–7, 10, 14, 15). It is well-known that the male: female ratio of intracranial meningiomas is 1:2 (22). In contrast, our cohort present no significant gender predilection. In our case series, the sex ratio (male: female) was ~1:1. This may be due to the high proportion of WHO grade II and III (non-benign) meningiomas (41.2%) in this series. Non-benign meningiomas are more common in males (22). This may be why there was a trend toward increased recurrence in men in the univariate analysis (*P* = 0.097). Furthermore, it has been demonstrated that patients with non-skull base lesions are more likely to have non-benign meningiomas (27 vs. 12%, *P* < 0.001) (23). Such a higher proportion of non-benign meningiomas in the cohort may be related to more recurrent cases included. In our case series, the proportion of recurrent tumors that had initially been treated elsewhere was 58.8%.The reason for such a high proportion of high-grade meningiomas deserves further research.

As reported by Taro Shimono et al. (24), the most common site invaded by skull base meningiomas is the orbit, and cervical extension is rare, occurring in only 1.4% of all cases of intracranial meningioma. In our cohort, the most common site of tumor invasion was the orbit (88.2%), and thus the most common symptoms were ophthalmic symptoms, which is consistent with what has been previously reported (3, 4, 15). Among these symptoms, proptosis is the most common clinical sign of meningioma with orbital extension. Proptosis can be explained either by the growth of an intraorbital tumor, the osseous invasion of a tumor in the orbital walls, or the reduction of venous drainage from the orbit on account of dural infiltration at the level of the superior orbital fissure (SOF) (7).

Heterogeneous enhancement was found on contrast-enhanced MRI in 10 cases (29.9%) (Figures 7A–C). Heterogeneous enhancement could be due to the existence of necrosis and cystic degeneration. On account of the rapid proliferation rate, the central area of the tumor often has insufficient blood circulation, resulting in ischemic necrosis or cystic degeneration (25). The necrotic area is usually larger in more invasive tumors (25). In the study, bone destructive absorption accounts for 36.4% of the bone changes in the skull base, which is much higher than that reported in the literature (6, 8). And that may be related to the higher pathological grade and poor biological behavior of the tumor. Some authors have suggested that the finding of hyperostosis of the cranial base represents true invasion of the bone (6, 10–12, 14). In conclusion, the clinical and radiological features of skull base meningiomas with extracranial extensions can be summarized as “6M”: more often in males, more recurrent and non-benign cases, more orbital extension, more heterogeneous enhancement, and more bone destruction.

### Multidisciplinary Cooperation and Individual Surgical Strategies

Although GTR is always our primary goal, this could be tempered if the tumor involves crucial neurovascular structures. In this cohort, the total resection rate is not so high (55.9%) due to more extensive cavernous sinus involvement cases (14/34) and more recurrent cases (20/34). We advocate that skull base meningiomas with both extra- and intra-cranial extensions should be surgically treated by a multidisciplinary skull base team. The team members include neurosurgeons, head & neck surgeons, plastic and reconstructive surgeons. Patients with such special meningiomas usually need staged operations without multidisciplinary cooperation. In this study, all cases received one-stage surgery through multidisciplinary cooperation, which alleviated the suffering and economic burden related to staged surgery. In addition, STR or PR of tumor should be followed by adjuvant RT, especially for the recurrent or non-benign cases. So, multidisciplinary cooperation is crucial for the management of skull base meningioma with extracranial extensions.

#### Individual Surgical Approach

Skull base meningiomas with extracranial extensions originate in different site at the skull base, and different intra- and extracranial vital structures involved, so an individual approach should be applied for the tumor resection. We have summarized eight approaches applicable for the treatment of these special entities, as outlined below. (a) The Derome approach (Figure 1) is usually used for tumors of anterior cranial fossa extending into the nasal cavity and paranasal sinus. We can gain access to the anterior skull base, the medial part of the maxillary sinus, and the nasal cavity directly through this approach. Blind spots underneath the orbits are the limitation of this approach, but increased visualization is facilitated by the additional use of endoscopy (9). (b) The endoscopic endonasal approach (Figure 6) can be used for tumors of midline cranial base extending into the nasal cavity and paranasal sinus. The endoscopic endonasal approach has many advantages, such as the lack of external incisions, reduced brain retraction, direct access to the tumor under the midline cranial base and an acceptable complication profile in contemporary series. Due to the development of this approach, the lateral rhinotomy gradually faded from view. Many authors have indicated that a purely or additional endoscopic endonasal approach is feasible and effective for the resection of anterior cranial base meningiomas with extracranial involvement in selected cases (3, 4, 15, 26). Almeida et al. (13) indicated that the transorbital endoscopic eyelid approach is a novel minimally aggressive option for the resection of sphenoorbital meningiomas with predominant hyperostosis. (c) The frontotemporal approach, possibly in combination with additional orbital or zygomatic osteotomy (Figure 2), is used for anterolateral cranial base meningiomas invading sphenoid wing, petrous bone, orbit or fossae temporalis. The effectiveness of frontotemporal approach for excision of purely sphenoorbital meningiomas has been demonstrated by many reports (6–8, 11, 12, 14). (d) The maxillary swing approach (Figures 4H–L) is used for giant middle cranial fossa meningiomas extending into the infratemporal and pterygopalatine fossae, especially for those with heavy calcification and fibrosis. The advantages include wide tumor exposure, en bloc resection and less blood loss during operation (27). (e) The extended middle cranial fossa approach is suitable for meningiomas of which the major part locates in the middle skull base while a minor part extends to the infratemporal or pterygopalatine fossae. Middle cranial base meningiomas with both the infratemporal fossa and the posterior fossa extensions can be removed via this approach (Figure 3). (f) A combined craniofacial approach (Figures 4A–G) is only used for tumors with widely intracranial and extracranial involvement that cannot be removed by a single transcranial or transfacial approach. We agree with Emel Avci et al. (28) that the Barrow classification is a effective and simple system to understand the surgical anatomy better and refine the techniques for performing complex craniofacial approaches. However, the transfacial approach can be replaced by the endoscopic endonasal approach in selected cases. (g) The combined craniocervical approach (Figure 5) is used for dumbbell shaped jugular foramen tumors with cervical extensions. The vessels and nerves in the neck needed to be recognized and protected first, followed by extracranial tumor removal and exploration of jugular foramen. The intracranial and jugular foramen tumors were then removed by neurosurgeons via a suboccipital craniotomy. The advantages of this approach are that it is beneficial for protecting neurovascular structures and such an incision can provide an adequate vascularized muscle flap to reconstruct the skull base (17). (h) The undefined approach (Figures 7A–G). For tumors with extensive skull base invasion or skin ulceration, when existing surgical approaches cannot be applied, an undefined approach can be used to achieve radical tumor resection. And the large defect after tumor or ulcerative skin resection was reconstructed with the assistance of head and neck plastic surgeons.

#### Reconstruction of the Skull Base

The reconstruction of skull base mainly includes bone reconstruction and soft tissue reconstruction. The specific reconstructive procedure was selected based upon several key factors, including the location of the defect, the defect size, the tissue involved and whether post-operative radiotherapy is needed. Bony defects left after resection of skull base tumors rarely require hard support. Considering that some patients have received radiotherapy or need post-operative radiotherapy, we believe that soft tissue reconstruction is far more important than bone reconstruction. At present, it is controversial whether the orbital wall is reconstructed after orbital tumor resection. For the resection of sphenoorbital meningiomas in this group, the orbital rim was kept intact as much as possible for aesthetic reasons, so reconstruction of the orbit was unnecessary. There exists a high risk of osteonecrosis and bony resorption when an autologous free bone is used in orbital repair (29). We suggest using titanium mesh and vascularized soft tissue to remedy this problem. Oya et al. (6) suggested not attempting to radically resect portions of the tumor beyond the periorbita. They believe that there is no need to reconstruct the orbit if the periorbita remains attached to the orbital rim. Shrivastava et al. (8) advocated that if more than one orbital wall is removed, extensive reconstruction of the orbit is necessary to avoid extraocular muscle fibrosis, pulsating enophthalmos, and post-operative meningoceles.

Soft tissue repair mainly includes the following situations. (a) For small skull base defects after resection of the tumor by endoscopic approach or transfacial approach, autologous fat packing and nasoseptal flap can be used to repair the defect. Several reports have indicated that the use of pedicled septal flap and free fat grafts is an effective and safe technique for repairing skull base defects (30–33). (b) For a moderate skull base defects (generally no more than 4 cm in maximum diameter) left after transcranial approach, the dura mater can be repaired with autologous fascia and covered with adjacent pedicled myofascial flap. For example, the pedicled frontalis myofascial flap was used in the anterior approach (Figure 1E), the pedicled temporalis myofascial flap was used in the lateral approach, and the pedicled sternocleidomastoid myofascial flap was used in the combined craniocervical approach. It should be noted to avoid damaging the blood supply of the flap during operation. Feng et al. (34) also recommended temporalis muscle flap as a good choice for reconstruction of the lateral skull base. It has been reported that some flaps, such as the side-door temporoparietal fascia flap and the helmet-visor pericranial flap, can be used as a new option for skull base reconstruction (35, 36). (c) When large defects remain following tumor ablation (generally more than 4 cm in maximum diameter) or there is no available regional pedicled myofascial flap or skin defect, free flap transplantation can be used for repair. The free flap provides large and well-vascularized tissue, so it can effectively fill the dead space. And because the free flap does not have the attachment of a pedicle, it can be designed and placed in the desired position. Aksu et al. (37) have summarized six different types of free flaps used for cranial base reconstruction including anterolateral thigh flap, vertical rectus abdominis flap, radial forearm flap, fibula osteocutaneous flap, iliac osteocutaneous flap and tensor fasciae latae flap. The most commonly used flap in our center is the anterolateral thigh flap (Figures 7E,F). The superficial temporal artery, the facial artery or the occipital artery are the common recipient vessels. The flap can be harvested without changing intraoperative positioning, which allows both the recipient-site team and the donor-site team to operate simultaneously. Some studies have confirmed the effectiveness of the anterolateral thigh flap in repairing large skull base defects (29, 38). However, it has been reported that the failure rate of free flap repair is 2–9% (38). Advanced age and cardiovascular disease proved to increase the risk of flap ischemia (38). In the group, there is a patient with high risk of thrombosis who developed both necrosis of the flap and cerebral infarction after operation. After anticoagulatant therapy and repeated flap transplantation, hemiplegia left by cerebral infarction was improved and the incision healed at the time of discharge. Therefore, we suggest that the risk of thrombosis should be assessed before flap transplantation and anticoagulatant therapy should be started as early as possible in high-risk patients.

### Recurrence and Parameters Associated With Prognosis

In the literature, recurrence rates of 8–56.3% have been reported in several series (5, 7, 8, 39–41). In our series, 12 of 34 patients (35.3%) developed progression or recurrence during the median follow-up period of 31 months. The Kaplan-Meier univariate analysis showed a significantly decreased time to recurrence in patients with histological risk factors (higher WHO grade or Ki-67 LI) or NGTR. In addition, multivariate analysis revealed that adjuvant RT was a prognostic factor of PFS.

#### EOR and Adjuvant RT

The EOR remains a key factor in reducing early recurrence, as has been reported in previous studies (1, 5–7, 20, 41, 42). After the complete resection of cranial base meningiomas, the recurrence rate ranges from 20 to 22% at 10 years, and the recurrence rate of incomplete resection is significantly higher, ranging from 55 to 74% at 10 years (9). In our series, there were 4 cases (21.1%) of recurrence with GTR (4/19) and 8 cases (57.1%) of recurrence with NGTR (8/14). So, it's still our primary goal to achieve a GTR. Many authors have suggested that the combination of NGTR and adjuvant RT increased PFS with efficacy similar to that of GTR alone (1, 41, 43). Thus, NGTR followed by adjuvant RT can sometimes replace GTR. It is well-known that adjuvant RT is routinely used for WHO-III meningiomas and WHO-II meningiomas with NGTR. However, the role of adjuvant RT remains controversial for WHO-II meningiomas with GTR. Some authors have recommended adjuvant RT for WHO-II meningiomas regardless of the EOR (41, 44). In contrast, some authors do not support such aggressive use of adjuvant RT, only for tumors with NGTR (45, 46). We support the latter view. For tumors with GTR and Ki-67 <5, active surveillance is enough. But for the tumors with GTR while Ki-67 ≥ 5, it should also be candidates for RT.

#### Histological Grade and Biological Markers

The proportion of non-benign meningiomas in the skull base reported in the present study is significantly higher than that reported in the aforementioned literature (1, 5, 20, 43). This could be due to more recurrent cases included, more cases of extensive skull base destructive absorption, and limited cases with selection bias. Associations between histological grades and PFS have been reported in the literature, with recurrence rates of 7–25, 29–52, and 50–94% for WHO grades I, II, and III, respectively (1). The recurrence rates in our cohort were 21.1%, 58.5%, and 50.0%, respectively.

Nevertheless, meningiomas of the same pathological grade do not always show the same biological activity. Therefore, it is important to identify a useful marker for predicting the risk of tumor progression. The Ki-67 LI was examined in addition to the histological grade in our study. Many studies have shown that the recurrence of meningiomas is associated with the increased Ki-67 LI, in agreement with the present study (1, 19, 20, 47). In our cohort, adjuvant RT and the EOR showed different associations with PFS between the two groups (Ki-67 ≥ 5 or Ki-67 <5) (Figure 9), which may indicate that the effect of adjuvant RT on recurrence in patients with Ki-67 LI ≥ 5 was greater than that of the EOR; the conclusion was opposite in the other group. Thus, it enlightens us that skull base meningiomas with extracranial extensions with a high Ki-67 LI should be candidates for adjuvant RT to reduce recurrence. In the management of these complex tumors, Assessment of the Ki-67 LI should be recommended to determine subsequent treatment. However, this factor has not been included in the WHO diagnostic criteria for high-grade meningiomas.

### Treatment Algorithms

**Figure 10:**
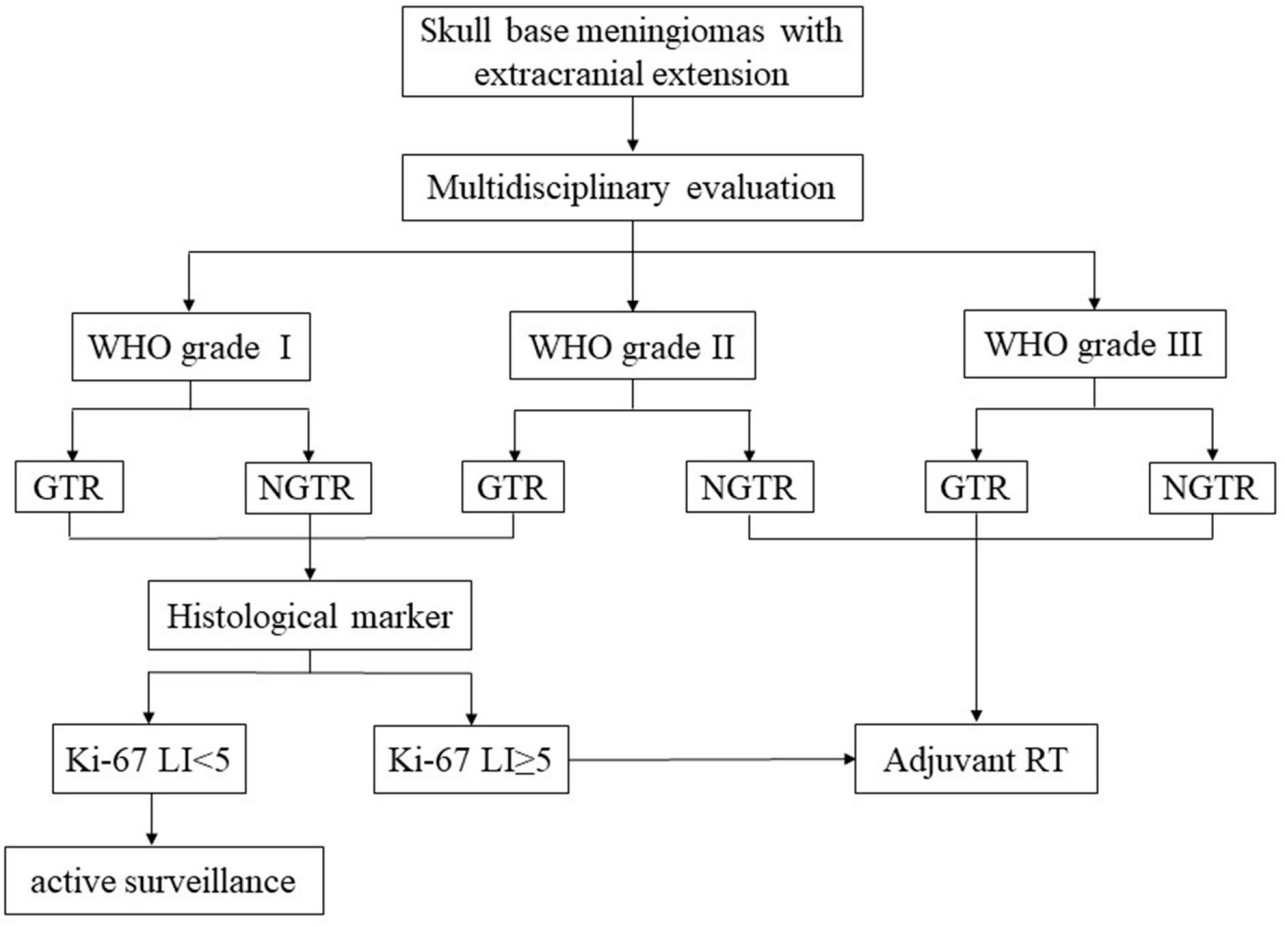
Treatment algorithms for skull base meningiomas with extracranial extension.

Based on the evolving literature and our institutional data on the management of these rare meningiomas, we suggest those algorithms for the treatment of these complex tumors (Figure 10).

## Conclusions

Skull base meningiomas with extracranial extensions are a relatively rare group of meningiomas that has a higher proportion of males and high histological grade compared with intracranial meningiomas. There are a low total resection rate and a high recurrence rate as different intracranial and extracranial structures involved by tumors. So, multidisciplinary collaboration, which may involve neurosurgery, head and neck/otolaryngology, plastic surgery and radiation oncology, is beneficial for the surgical management for these tumors. An individualized surgical strategy should be designed for each patient. Maximal tumor removal with minimal surgical morbidities remains the optimal treatment to minimize local recurrence. STR or PR followed by adjuvant RT is a reasonable strategy when radical resection is unavailable. RT for residual tumors should be considered in patients with histopathological risk factors, such as a high histological grade or Ki-67 ≥ 5. Active surveillance could be considered for patients without these risk factors.

## Data Availability Statement

All datasets generated for this study are included in the article/Supplementary Material.

## Ethics Statement

The study was approved by the Cancer Hospital, Chinese Academy of Medical Science and Peking Union Medical College Research Ethics Committee. Written consent from patients that are identifiable from the images have been obtained.

## Author Contributions

JW conceived the idea. HL and FZ collected the data. HL analyzed the data and drafted the manuscript. All authors participated in the surgical procedure.

## Conflict of Interest

The authors declare that the research was conducted in the absence of any commercial or financial relationships that could be construed as a potential conflict of interest.
