# Supplementary material for: Clinial Features, Individualized Treatment and Long-Term Surgical Outcomes of Skull Base Meningiomas With Extracranial Extensions
Source: Front Oncol. 2020 Jun 30;10:1054. doi: 10.3389/fonc.2020.01054 (PMC7340145; doi:10.3389/fonc.2020.01054)
Supplement: Supplementary file 1 [file Table_1.DOCX]

**Supplementary table.** Details for clinical data of 34 patients with skull base meningiomas with extracranial extensions.

| Patients | Age/ sex | Recurrent lesion? | Extracranial extension^a^ | Surgical approach^b^ | EOR | Pathology/ ki-67 LI(%) | Complication | Adjuvant RT (Y or N ) | Recurrence(Y or N) |
| --- | --- | --- | --- | --- | --- | --- | --- | --- | --- |
| 1 | 23/M | Y | Orbit, PNS, NC | CCFA | NGTR | WHO-I/ 2 | CSF leakage | Y | Y |
| 2 | 37/F | N | Orbit | FTA | NGTR | WHO-I/ 8 | NO | Y | N |
| 3 | 63/F | Y | Orbit, PNS | CCFA | GTR | WHO-I/ <5 | NO | Y | N |
| 4 | 26/M | N | Orbit, PNS | DA | GTR | WHO-I/ >5 | NO | N | Y |
| 5 | 58/F | N | Orbit, PNS | EEA | NGTR | WHO-III/ 20 | NO | N | Y |
| 6 | 38/M | Y | Orbit, PNS | FTA | NGTR | WHO-II/ >5 | NO | Y | Y |
| 7 | 63/F | N | Orbit | FTA | GTR | WHO-I/ <5 | NO | N | N |
| 8 | 14/F | N | Orbit, PNS | DA | GTR | WHO-I/ <5 | NO | N | N |
| 9 | 47/M | Y | Orbit, NC | DA | GTR | WHO-II/ >5 | NO | Y | Y |
| 10 | 48/F | N | Orbit | FTA | GTR | WHO-I/ <5 | NO | N | N |
| 11 | 59/M | Y | Orbit, PNS, ITF, PPF | MSA | NGTR | WHO-I/ <5 | NO | Y | N |
| 12 | 48/M | Y | Orbit | FTA | NGTR | WHO-II/ 5-10 | subcutaneous hydrops | Y | N |
| 13 | 72/F | N | Orbit | FTA | GTR | WHO-II/ <5 | NO | N | N |
| 14 | 22/F | N | Orbit, PNS, NC | DA | GTR | WHO-I/ <5 | NO | N | N |
| 15 | 46/M | Y | Orbit, PNS, NC | DA | GTR | WHO-I/ <5 | NO | Y | N |
| 16 | 48/F | N | ITF, PPF | CCFA | NGTR | WHO-II/ 10 | NO | N | Y |
| 17 | 33/M | Y | Orbit, PNS, NC | DA | GTR | WHO-II/ >5 | NO | N | Y |
| 18 | 39/F | N | Neck, PPS | CCCA | NGTR | WHO-I/ 2 | NO | Y | N |
| 19 | 53/M | Y | Orbit, PNS, ITF, PPF | MSA | GTR | WHO-II/ 20 | CSF leakage, intracranial infection | Y | N |
| 20 | 52/F | Y | Orbit, PNS, NC | DA | GTR | WHO-I/ 5 | NO | Y | N |
| 21 | 55/M | Y | Orbit, PNS | FTA | GTR | WHO-I/ 5 | subcutaneous hydrops | N | N |
| 22 | 43/M | Y | Orbit, PNS, ITF | MCFA | GTR | WHO-III/ >5 | NO | NA^c^ | NA |
| 23 | 59/F | Y | Orbit, ITF | CCFA | GTR | WHO-I/ <5 | NO | N | N |
| 24 | 62/M | N | Orbit, PNS, NC | DA | GTR | WHO-I/ <5 | intracranial infection | N | N |
| 25 | 47/M | Y | Orbit, ITF | FTA | GTR | WHO-I/ >5 | NO | N | Y |
| 26 | 46/F | N | Neck | CCCA | GTR | WHO-I/ <5 | NO | N | N |
| 27 | 50/M | Y | Orbit | FTA | NGTR | WHO-I/ <5 | brain stem dysfunction | N | NA |
| 28 | 46/M | Y | Orbit, PNS, NC | UDA | NGTR | WHO-II/ >5 | NO | N | Y |
| 29 | 34/F | N | Neck | CCCA | NGTR | WHO-I/ <5 | cranial nerve deficit | Y | Y |
| 30 | 60/M | Y | Orbit, PNS, PPF | MSA | NGTR | WHO-II/ 2-3 | NO | N | N |
| 31 | 54/F | Y | Orbit, PNS, NC, ITF, PPF, ear | UDA | GTR | WHO-II/ 15 | cerebral infarction, skin flap necrosis | N | N |
| 32 | 41/F | Y | Orbit | FTA | NGTR | WHO-I/ 2 | cranial nerve deficit | N | N |
| 33 | 68/F | N | Orbit | FTA | NGTR | WHO-II/ 30 | NO | N | Y |
| 34 | 50/M | Y | Orbit, PNS, NC | EEA | NGTR | WHO-II/ 10 | cranial nerve deficit | N | Y |

^a^ PNS, paranasal sinus; NC, nasal cavity; ITF, infratemporal fossa; PPF, pterygopalatine fossa; PPS, parapharyngeal space.

^b^ CCFA, the combined craniofacial approach; FTA, the frontotemporal approach; DA, the Derome approach; EEA, the endoscopic endonasal approach; MSA, the maxillary swing approach; CCCA, the combined craniocervical approach; MCFA, the middle cranial fossa approach; UDA, the undefined approach.

^c^ NA, not available.
